# The Impact of Shame, Self-Criticism and Social Rank on Eating Behaviours in Overweight and Obese Women Participating in a Weight Management Programme

**DOI:** 10.1371/journal.pone.0167571

**Published:** 2017-01-20

**Authors:** Cristiana Duarte, Marcela Matos, R. James Stubbs, Corinne Gale, Liam Morris, Jose Pinto Gouveia, Paul Gilbert

**Affiliations:** 1 Cognitive and Behavioural Centre for Research and Intervention, University of Coimbra, Rua do Colégio Novo, Apartado, Coimbra, Portugal; 2 College of Life and Natural Sciences, University of Derby, Derby, United Kingdom; 3 Nutrition and Research Department, Slimming World, Somercotes, Alfreton, Derbyshire, United Kingdom; 4 Mental Health Research Unit, Kingsway Hospital, Derby, United Kingdom; Hospital Universitari de Bellvitge, SPAIN

## Abstract

Recent research has suggested that obesity is a stigmatised condition. Concerns with personal inferiority (social rank), shame and self-criticism may impact on weight management behaviours. The current study examined associations between social comparison (shame, self-criticism), negative affect and eating behaviours in women attending a community based weight management programme focused on behaviour change. 2,236 participants of the programme completed an online survey using measures of shame, self-criticism, social comparison, and weight-related affect, which were adapted to specifically address eating behaviour, weight and body shape perceptions. Correlation analyses showed that shame, self-criticism and social comparison were associated with negative affect. All of these variables were related to eating regulation and weight control (*p* < 0.001). Path analysis revealed that the association of shame, hated-self, and low self-reassurance on disinhibition and susceptibility to hunger was fully mediated by weight-related negative affect, even when controlling for the effect of depressive symptoms (*p <* 0.050 to *p* < 0.010). In addition, feelings of inadequacy and unfavourable social comparisons were associated with higher disinhibition and susceptibility to hunger, partially mediated through weight-related negative affect (*p* = 0.001). These variables were negatively associated with extent of weight loss during programme attendance prior to the survey, while self-reassurance and positive social comparisons were positively associated with the extent of weight loss prior to the survey (*p* < .050). Shame, self-criticism, and perceptions of inferiority may play a significant role in self-regulation of eating behaviour in overweight people trying to manage their weight.

## Introduction

Projected obesity trends are well documented [1–4], escalating [5] and account for a significant proportion of health costs in Europe [6] and the US [7]. While prevention is preferable to cure, the majority of Western adults are already overweight or obese [8], which emphasises the need to provide self-management solutions to prevent further weight gain and promote sustained weight loss [2, 9]. Governments are calling for consumers to focus on the proactive prevention of avoidable disease by taking more responsibility for their own health through the adoption of healthier lifestyles, improved diets, increased physical activity and managing their own weight [10, 11]. However, many people are enmeshed in an “obesogenic” environment that facilitates excess energy intake and low levels of energy expenditure [12–15]. Self-management of behaviour may also be influenced by emotion regulation, i.e., the processes whereby individuals seek to manage emotional sates [16, 17]. It is not clear if emotion regulation helps or hinders self-management of weight. Part of the reason for this is that food can have both positive and negative effects on emotive and reasoned thoughts and actions and weight management can also have a large emotional dimension. Food is a powerful source of pleasure and reward [18–21]. Also, eating may serve the function of temporarily reducing negative affect and thus regulating aversive emotions [22, 23]. This may have consequences for the enactment of planned, reasoned behavioural pathways to manage weight. Overweight and obese people commonly experience stigma, which enhances psychosocial stress and negatively impacts on physical and mental wellbeing [24–26]. Stigma can impact on shame, self-criticism and unfavourable social comparisons, creating feelings of inferiority and inadequacy in relation to others [27].

The process of stigmatisation of body shapes means that physical appearance can affect the way people feel. Weight stigma can become associated with feelings of inferiority, shame and self-criticism [27–29]. Shame and self-criticism are associated with depression [30], body image dissatisfaction [28, 31–35], binge eating [36–39] and obesity [40]. Shame-based self-criticism may undermine self-regulation of eating behaviour.

This study explored, through an online survey, the associations between self-evaluative processes (shame, self-criticism, social comparison), weight-related affect and depressive symptoms in people attending a community-based weight management programme. Further analysis controlled for depressive symptoms and then examined the relationship between self-evaluative processes and (i) control of eating behaviour (measured by disinhibition and susceptibility to hunger) and (ii) historical weight change during programme attendance prior to the time of the survey.

## Materials and Methods

### Subjects

Participants were 2,236 women attending a community based weight management programme focused on behaviour change. Table 1 gives participants’ age, height, weight and BMI when they started with the programme, along with their weight and BMI at the time they completed the survey, weight change (*p* < 0.001) and BMI change (*p* < 0.001) between starting with the programme and the point of survey. Time taken to reach survey weight was the number of days from joining the programme until the date of the survey. Nineteen point one percent had a BMI between 25–30 kg/m^2^, 33.5% between 30–35 kg/m^2^, 23% between 35–40 kg/m^2^ and 24.4% > 40 kg/m^2^. Compared to average participants in the programme these women lost more weight and attended for longer. Start height, weight and age were similar to average participants of the programme [41].

**Table 1:** Characteristics of study participants.

	*N*^1^	*M*	*SD*
Age	2236	41.71	12.34
Height (m)	2236	1.65	0.07
Start Weight (kg)	2236	95.61	18.73
Start BMI (kg/m^2^)	2236	35.28	6.49
Weight at survey (kg)	2089	85.65	17.46
BMI at Survey (kg/m^2^)	2089	31.62	6.10
Weight change (kg)	2089	-9.98**	8.80
BMI Change (kg/m^2^)	2089	-3.68**	3.22
Weight change (%)	2089	-10.19%**	8.07%
Time taken to reach survey weight (days)	2236	274.44	388.76
	*N*	%	
Lost weight	2055	91.4	
Gained weight	27	1.3	
Maintained weight	7	0.3	

Note:

^1^ For 147 participants (7%) weight data was not provided at the beginning of the survey.

** Denotes statistical significance at *p* < 0.001.

### Procedure

The commercial weight management organisation, Slimming World (www.slimmingworld.com), meets the NICE best practice criteria [3] to help adults adopt lifestyle behaviour changes to reduce weight, prevent weight gain and support long-term weight maintenance. The programme and approach to behaviour change and weight management are described elsewhere [9, 42, 43].

After being approved by the University of Derby Ethics Committee, this study was advertised on the Slimming World members’ website. The advertorial directed potential participants to a website designed specifically for this project, which provided detailed information about the study and contact details for the research team at the University of Derby to answer any specific questions relating to the study. Those wishing to take part were asked to indicate their consent by clicking the appropriate button on the website. Once consent had been obtained participants were directed to a link to the questionnaires, which were completed online.

The questionnaire (available as online supplementary material S1 File) consisted of questions regarding age, height, level of activity, date of birth, duration of membership, time taken to reach current weight and time at current weight. Duration of membership, time taken to reach current weight and time at current weight, were computed by assuming linearity and taking the mid-point of each time category (i.e., 6 months or less as 3 months, 6–12 months as 9 months, 12–18 months as 15 months, 18 months-2 years as 21 months, 2–3 years as 30 months, more than 3 years as 48 months). The remainder of the questionnaire took the form of Likert scales asking questions about shame, self-criticism and social comparison, negative and positive affect related to body weight and shape, and eating, depressive symptoms, and eating behaviours, as described below. The questionnaire was constructed and administered using Checkbox v4.4-Web Survey Software- 2007, Prezza Technologies, Inc. The specific scales used in the current study are described in the supplementary material (S1 File).

### Materials

#### Weight-focused external shame scale (WFES)

This scale was adapted from the Other as Shamer Scale [44, 45]. The scale consists of 18 items, which participants are asked to rate in terms of the frequency with which they make certain evaluations about how others judge them based on their weight, body shape and eating (0 = ‘Never’ to 4 = ‘Almost always’). In the original study the scale showed high internal consistency with a Cronbach’s alpha of 0.92.

#### Weight-focused self-criticising/self-reassuring scale (WFSCRS)

This 22-item scale is derived from the Forms of Self-Criticising/Attacking and Self-Reassuring Scale [46]. Participants rate each statement on a five-point scale (0 = ‘Not at all like me’ to 4 = ‘Extremely like me’). Subscales include inadequate self, which regards a sense of feeling internally put-down and inadequate; hated self, which involves a sense of self-dislike and aggressive/persecutory desires to hurt the self; and reassured self, which refers to an ability to be encouraging and concerned for self when things go wrong. The original scale has good reliability with Cronbach’s alphas of 0.90 for inadequate self, 0.86 for hated self, and 0.86 for reassured self [46].

#### Weight-focused social comparison scale (WFSCS)

This 11-item scale measures judgements of how a person compares him/herself with others in terms of general competencies (inferior-superior), attractiveness (undesirable—more desirable) and how well the person thinks he/she ‘fits in’ (outsider-insider). This scale uses a semantic differential methodology with bipolar constructs on a 1–10 scale. Lower scores point to feelings of inferiority and general low rank self-perceptions. The scale has been found to have good reliability, with Cronbach’s alphas of 0.88 and 0.96 in clinical populations and 0.91 and 0.90 with student populations [47]. For this study the instructions were changed to focus on weight, body shape or eating.

#### Weight-focused feelings scale (WFFS)

This is an 11-item scale measuring positive and negative feelings in relation to body weight, body shape and eating. Participants were asked to rate on a 4-point scale (‘Not like me’ to ‘Extremely like me’) their responses to a series of statements about their current feelings linked to body weight. Exploratory and confirmatory factor analyses of this measure showed that this scale shows good psychometric properties with a robust two-factor structure: negative weight-focused feelings (8 items; e.g., ‘I feel depressed and down’); and positive weight-focused feelings (3 items; e.g., ‘I am quite happy in myself’).

#### Depression, anxiety and stress scale (DASS-21)

This 21-item scale measures depressive, anxiety and stress symptoms [48]. Participants were asked to rate how much each statement applied to them over the past week, on a 4-point scale (‘Did not apply to me at all’ to ‘Applied to me very much, or most of the time’). The subscales presented Cronbach’s alphas of 0.94 for depression, 0.87 for anxiety and 0.91 for stress [49].

#### The three factor eating questionnaire (TFEQ)

The TFEQ is a 51-item questionnaire designed to measure three cognitive and behavioural dimensions of eating [50]. The TFEQ includes three subscales: restraint, which measures the tendency to restrict food intake to control body weight and shape; disinhibition, which assesses episodes of loss of control over eating; and susceptibility to hunger, which measures subjective perceptions of hunger and food cravings [51]. In the original study the scale revealed Cronbach’s values of 0.93 for the subscale Restraint, 0.91 for the subscale Disinhibition, and 0.85 for the subscale Hunger.

#### Height and weight

The weight management programme support groups are located in a variety of local venues at different times and days of the week, making the groups widely accessible for members of the community. The majority of participants access the groups through self-referral and pay weekly (£4.95) to attend their chosen group. It is an open programme with no fixed duration of membership. Participants can join, leave and re-join as they wish for any length of time and support groups are available week-by-week through the year. As part of the weight management programme height is recorded at the point of joining and weight is recorded weekly during programme attendance. Height was self-reported to the nearest 0.5 cm. Participants were weighed in light clothing on scales with a precision of ± 0.23 kg (SECA bespoke model). Accuracy is ensured by calibration against standard weights, during routine service and scales are checked for notable drift weekly in use. The same calibrated scales were used each week at a given group to record weight and weight change. Weights reported for the time of survey were < 10 days of the survey date.

#### Statistical analysis

Data analyses were conducted using PASW (version 18 SPSS, Chicago Inc.) and AMOS (version 18, SPSS Inc, Chicago, IL).

Pearson correlation coefficients were calculated to explore the correlations between the study variables. Multiple regression analyses were conducted to explore the contribution of study variables to the prediction of depressive symptoms and weight-related negative affect [52].

A mediation analysis was then performed, to test the impact of weight focused external shame (WFES), weight focused self-criticism (WFFSCRS) and weight focused social comparison (WFSCS); [independent, exogenous variables] on disinhibition and susceptibility to hunger (TFEQ); [dependent, endogenous variables] with weight-related negative affect (WFFS) as a mediator, controlling for the effect of depressive symptoms (DASS-21). A final path analysis was conducted examining the direct and indirect effect of these variables on the extent of weight loss during participation in the programme prior to the point of survey (BMI change; dependent, endogenous variable).

A path analysis using the Maximum Likelihood method was carried out to test for the relationships described above. This technique is a special case of structural equation modelling (SEM) and considers hypothetic causal relations between variables, controlling for error [53, 54]. There was no severe violation of normal distribution (with skewness values ranging from -0.37 (disinhibition) to 1.19 (DASS-21 depression), and with kurtosis values ranging from -0.95 (susceptibility to hunger) to 0.83 (DASS-21 depression). The significance of direct, indirect and total effects was assessed using *χ*^2^ tests. Bootstrap re-sampling was further used to test the significance of the mediation paths, using 1000 bootstrap samples and 95% confidence intervals (CIs; [54]). The data set used for analysis in the present study can be found in the supplementary materials S1 Dataset.

## Results

Mean (*SD*) weight change, since joining the programme, was -9.98 (8.80) kg, BMI change was -3.68 (3.22) kg/m^2^ and per cent weight change was -10.2 (8.1)% (all *p* < 0.001) indicating that this group of participants lost a significant amount of weight during programme attendance up to the point of survey. Mean BMI was 31.6 kg/m^2^ at the time of survey.

The scales, which were adapted to focus dimensions of weight and eating relevant to this population (WFES, WFFSCRS, WFSCS), presented higher mean values than those found with the original versions in the general population [44, 46, 47] and similar to those found in clinical samples with eating disorders [27, 28, 35]. Participants showed higher levels of negative affect related to weight and eating than of positive affect. Depressive symptoms scores were similar to those found in the general population [48]. Mean values for disinhibition and susceptibility to hunger TFEQ subscales were comparable to those found in other studies conducted in similar samples [55, 56]. All scales revealed good to very good internal consistency (Table 2).

**Table 2:** Descriptive statistics and Cronbach’ alphas for study variables (N = 2 236).

	Cronbach’α	*M*	*SD*
WFES	.95	24.50	14.42
WFFSCRS_Inadequate Self	.86	19.16	8.15
WFFSCRS_Reassured Self	.84	16.38	6.31
WFFSCRS_Hated Self	.70	4.66	4.43
WFSCS	.95	53.53	18.40
WFFS_Negative Affect	.88	12.64	8.37
WFFS_Positive Affect	.79	5.13	3.45
TFEQ_ Disinhibition	.79	9.94	3.55
TFEQ_Susceptibility to hunger	.82	6.51	3.70
TFEQ_Dietary Restraint	.70	11.50	3.43
DASS21_Depression	.91	5.08	4.98

Note: WFES = Weight-focused External shame Scale; WFFSCRS = Weight-focused Self-Criticising/Self-Reassuring Scale; WFSCS = Weight-focused Social Comparison Scale; WFFS = Weight-focused Feelings Scale; TEFQ = Three Factor Eating Questionnaire; DASS21 = Depression, Anxiety and Stress Scale-21.

### Correlations

Table 3 gives the correlation matrix for the study variables. Results showed positive associations between depressive symptoms, external shame, self-criticism, and weight-related negative affect. Negative correlations were found between these variables and weight-related positive affect, favourable social comparisons and reassured self.

**Table 3:** Pearson’s correlations between the study measures (N = 2 236).

	1	2	3	4	5	6	7	8	9	10	11	12
1. BMI												
2. BMI_Change	-0.36**											
3. WFES	0.19**	-0.06*										
4. WFFSCRS_Inadequate Self	0.15**	-0.13**	0.64**									
5. WFFSCRS_Reassured Self	-0.14**	0.07*	-0.50**	-0.49**								
6. WFFSCRS_Hated Self	0.19**	-0.14**	0.62**	0.71**	-0.54**							
7. WFSCS	-0.22**	0.15**	-0.65**	-0.57**	0.59**	-0.56**						
8. WFFS_Negative Affect	-0.19**	-0.20**	0.63**	0.73**	-0.55**	0.70**	-0.62**					
9. WFFS_Positive Affect	-0.11**	0.28**	-0.49**	-0.55**	0.68**	-0.53**	0.63**	-0.63**				
10. TEFQ_Disinhibition	0.10**	-0.16**	0.31**	0.39**	-0.30**	0.33**	-0.38**	0.42**	-0.43**			
11.TEFQ_Susceptibility to Hunger	0.04**	-0.14**	0.28**	0.32**	-0.22**	0.29**	-0.29**	0.35**	-0.34**	0.62**		
12. TEFQ_Dietary Restraint	-0.10**	0.12**	-.07**	-.03	0.11**	-0.06**	0.17**	-0.09**	0.13**	-0.22**	-0.20**	
13. DASS21_Depression	0.13**	-0.10**	.61**	.57**	-0.50**	0.61**	-0.54**	0.67**	-0.51**	0.31**	0.27**	-0.07**

*Note*. Correlations for BMI_Change considered participants with BMI data (*n* = 2 089).

** *p* < .001.

* *p* < .05.

BMI = Body Mass Index; WFES = Weight-focused External shame Scale; WFFSCRS = Weight-focused Self-Criticising/Self-Reassuring Scale; WFSCS = Weight-focused Social Comparison Scale; WFFS = Weight-focused Feelings Scale; TEFQ = Three Factor Eating Questionnaire; DASS21 = Depression, Anxiety and Stress Scale-2.

Disinhibition and susceptibility to hunger were positively associated with external shame, self-criticism, negative social comparison, weight-related negative affect and depressive symptoms, and negatively linked to reassured self and weight-related positive affect. Dietary restraint showed weak positive correlations with reassured self, positive social comparison and weight-related positive affect.

Women with higher BMIs had higher external shame, self-criticism (especially self-hatred), were less self-reassuring, felt more inferior to others, had higher weight-related negative and lower positive affect. Weight loss was positively associated with favourable social comparisons, self-reassurance, and weight-related positive affect, and negatively related to self-criticism, weight-related negative affect, disinhibition, susceptibility to hunger and depressive symptoms.

### Multiple regression with social rank variables predicting depressive symptoms and weight-related affect

Two multiple regressions were conducted using external shame, self-criticism and social comparison to predict depressive symptoms and weight-related affect.

For depressive symptoms, the regression model explained 49% of the variance [*F* = 432.28, *p* < 0.001]. External shame (β = 0.25) and hated self (β = 0.25) emerged as the best global predictors, followed by inadequate self (β = 0.12), reassured self (β = -0.12) and social comparison (β = -0.10).

Regarding weight-related negative affect, the model explained 65% of the variance [*F* = 825.61, *p* < 0.001], and inadequate self (β = 0.35) emerged as the best global predictor, followed by hated self (β = 0.24), social comparison (β = -0.16), reassured self (β = -0.11) and external shame (β = 0.10).

The same model was tested to predict weight-related positive affect and explained 57% of the variance [*F* = 593.26, *p* < 0.001], with reassured self (β = 0.43) emerging as the best global predictor, followed by social comparison (β = 0.29), and in a negative direction inadequate self (β = -0.16). All coefficients were significant at *p* < 0.001.

### Weight-related negative affect as a mediator between social rank variables and eating behaviours

The hypothesized model was first tested through a fully saturated model with 54 parameters. The direct effects of depressive symptoms, external shame, hated self and reassured self on disinhibition and susceptibility to hunger were not significant. These non-significant paths were removed and the model recalculated. Results indicated an excellent model fit: [χ^2^_(8)_ = 15.134, *p* = 0.057; CMIN/DF = 1.892; CFI = 0.999; TLI = 0.997; NFI = 0.999; RMSEA = 0.020 [0.000 to 0.035], *p* = 1.000), according to recommended model fit indices [54].

**Fig 1:**
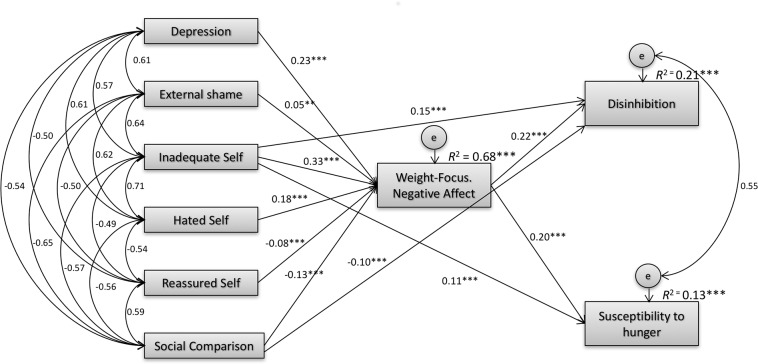
Path analysis for eating disinhibition and susceptibility to hunger. Mediation model with standardized estimates and *R*^2^ for weight-related negative affect, disinhibition and susceptibility to hunger. Model fit: χ^2^_(8)_ = 15.134 (*p* = 0.057); CMIN/DF = 1.892; CFI = 0.999; TLI = 0.997; NFI = 0.999; RMSEA = 0.020, CI = 0.000 to 0.035, *p* = 1.000). When the effect of depressive symptoms was controlled for, the impact of shame, hated self and reassured self on disinhibition and susceptibility to hunger was fully mediated by their effect on weight-related negative affect. Inadequate self and negative social comparison predict higher disinhibition and susceptibility to hunger directly and partially through increased weight-related negative affect. Note: Depressive symptoms = Depression, Anxiety and Stress Scale-21 Depression subscale; External Shame = Weight-focused External shame Scale; Inadequate Self, Hated Self, Reassured Self = Weight-focused Self-Criticising/Self-Reassuring Scale subscales; Social Comparison = Weight-focused Social Comparison Scale; Weight-Focus. Negative Affect = Weight-focused Feelings Scale Negative Affect subscale; Disinhibition, Susceptibility to hunger = Three Factor Eating Questionnaire subscales.

The model accounted for 68%, 21% and 13% of the variance in weight-related negative affect, disinhibition and susceptibility to hunger, respectively. Results showed that, when controlling for the effect of depressive symptoms, inadequate self had a direct effect on disinhibition (*b* = 0.063; *SE*_*b*_ = 0.012; *Z* = 5.147; *p* < 0.001) and susceptibility to hunger (*b* = 0.051; *SE*_*b*_ = 0.013; *Z* = 3.825; *p* < 0.001). Social comparison also had a negative direct effect on disinhibition (*b* = -0.031; *SE*_*b*_ = 0.005; *Z* = -6.464; *p* < 0.001) and susceptibility to hunger (*b* = -0.020; *SE*_*b*_ = 0.005; *Z* = -3.917; *p* < 0.001). Furthermore, mediation analyses suggested that external shame and hated self indirectly predicted greater disinhibition (respectively: β = 0.010, CI = 0.001 to 0.021, *p* = 0.029; β = 0.040, CI = 0.027 to 0.056, *p* = 0.001) and susceptibility to hunger (β = 0.009, CI = 0.001 to 0.019, *p* = 0.024; β = 0.037, CI = 0.024 to 0.053, *p* = 0.001), fully mediated through weight-related negative affect. Higher levels of reassured self predicted lower levels of disinhibition (β = -0.017, CI = -0.027 to -0.009, *p* = 0.002) and susceptibility to hunger (β = -0.016, CI = -0.025 to -0.008, *p* = 0.001) fully mediated through lower levels of weight-related negative affect. Inadequate self and social comparison also had an indirect effect on disinhibition (respectively: β = 0.071, CI = 0.052 to 0.094, *p* = 0.001; β = -0.029, CI = -0.042 to -0.019, *p* = 0.001) and susceptibility to hunger (β = 0.065, CI = 0.046 to 0.086, *p* = 0.001; *b* = -0.027, CI = -0.038 to -0.017, *p* = 0.001). Fig 1 presents the mediation model with regression coefficients standardized estimates and *R*^2^ for disinhibition, susceptibility to hunger and weight-related negative affect.

**Fig 2:**
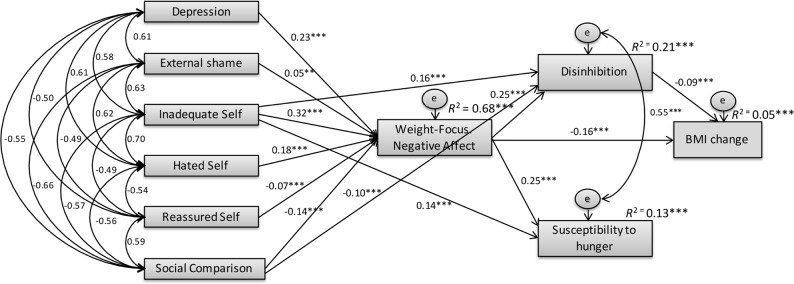
Path analysis for BMI changes. Mediation model with standardized estimates and *R*^2^ for weight-related negative affect, disinhibition, susceptibility to hunger and BMI changes. Model fit: χ^2^_(16)_ = 75.970 (*p* < 0.001); CMIN/DF = 4.748; CFI = 0.994; TLI = 0.984; NFI = 0.993; RMSEA = 0.042, CI = 0.033 to 0.052, *p* = 0.897).

Finally, we examined whether the proposed variables and mediators would have a significant effect on changes on BMI since starting the programme (*n* = 2 089), which was added in the model as the final outcome (Fig 2). Results indicated that the path between susceptibility to hunger and BMI change was nonsignificant (*p* = 0.165). This nonsignificant path was removed and results confirmed the adequacy of the model (χ^2^_(16)_ = 75.970 (*p* = 0.000); CMIN/DF = 4.748; CFI = 0.994; TLI = 0.984; NFI = 0.993; RMSEA = 0.042 [0.033 to 0.052], *p* = 0.897). Results indicated that, while controlling for the effect of depressive symptoms, shame (β = - 0.009,CI = -0.018 to -0.001, *p* = 0.029), hated self (β = -0.034, CI = -0.047 to -0.023, *p* = 0.002) and inadequate self (β = -0.074, CI = -0.090 to -.060, *p* = 0.002), had a negative indirect effect on BMI changes (i.e. weight loss), fully mediated by increased weight-related negative affect and higher eating disinhibition. Moreover, self-reassurance (β = 0.013, CI = 0.007 to 0.021, *p* = 0.002) and positive social comparisons (β = 0.035, CI = 0.026 to 0.046, *p* = 0.001) were positively associated with BMI changes (i.e. weight loss), and again their effects were fully mediated by lower negative affect and disinhibition. Negative affect presented a significant negative direct effect on BMI change (weight loss) (*b* = -0.059; *SE*_*b*_ = 0.009; *Z* = -6.889; *p* < 0.001), as well as a significant indirect effect, with eating disinhibition emerging as a significant mediator of the negative association between weight-focused negative affect and changes (decreases) in BMI (β = -0.022, 95% CI = -0.037 to -0.010, *p* = 0.002).

## Discussion

Current evidence suggests that core features of effective weight management interventions include self-regulatory behaviour change techniques such as self-monitoring (of weight and behaviour), relapse prevention, goal setting, and action plans for diet and physical activity [57–60]. However, for some there is also an emotional dimension to weight management. There is increasing evidence of the central role of shame, self-criticism, and negative social comparison in a wide range of psychopathological conditions [31, 32, 36, 46]. Self-evaluation and emotion regulation may also influence self-regulatory behaviours associated with weight management in some people. The current study examined associations between external shame, self-criticism, unfavourable social comparison, negative emotions about weight and dietary restraint, disinhibition and perceived hunger, in participating members of a weight management organisation that focuses on behaviour change.

In line with previous studies [31, 32, 36, 46], external shame, perceptions of social inferiority and self-criticism derived from weight-focused evaluations, a diminished ability to self-reassure and experience positive feelings in relation to weight and eating, were associated with depressive symptoms. Weight-related negative emotions were related to feelings of inferiority, external shame and self-criticism and a lack of self-reassurance, which is of interest in relation to potential mechanisms that may undermine control of eating behaviour. The relationships between negative self-evaluation and disinhibition and perceived hunger, suggest that shame, perceptions of inferiority in comparison to others and self-criticism may predispose people to poor self-regulation of eating behaviour within our current "obesogenic" and stigmatising environment [13–15, 25, 27].

Regression analysis confirmed the relationship between social rank variables, depressive symptoms and weight-related negative affect and identified salient predictors of weight-related negative and positive affect. Results suggested that negative self-evaluations (inferiority, perceived criticism or devaluation associated with weight status, along with self-criticism, have a significant effect on increased negative affectivity, which in turn, has been identified as an important predictor of difficulties in regulating eating behaviour [16, 22, 23].

Path analysis suggested that negative affect about weight is a mediator between external shame, social comparison, self-criticism, low self-reassurance, and indicators of difficulty in controlling eating behaviour (disinhibition and perceived hunger). These cross sectional data suggest that negative self-evaluation (shame, self-criticism including self-hatred, and low self reassurance) may be associated with negative feelings around one’s body weight and these emotions may translate into higher disinhibited eating behaviours and elevated predisposition to hunger. It would be important to confirm these associations in prospective intervention studies. These findings are consistent with previous studies indicating that perceptions of inferiority, shame and self-criticism play a significant role in the aetiology and persistence of disordered eating symptomatology [27, 28, 31, 35, 36, 38, 39]. The present study extends these findings by suggesting that these variables are also relevant to understand difficulties with self-regulation of eating behaviour in a large community sample overweight and obesity women.

Inclusion of weight change during participation of the programme prior to the survey confirmed that shame and self-criticism were negatively associated with extent of weight loss prior to the survey, while self-reassurance and positive social comparisons were positively linked with extent of weight loss prior to the survey. In the path analysis these effects appeared to be related to their effect on increased or decreased weight-focused negative affect and disinhibition/perceived hunger. In particular negative affect was negatively associated with degree of weight loss prior to the survey and was associated with higher eating disinhibition, which, in turn, was strongly linked with susceptibility to hunger.

It is important to emphasize the cross-sectional nature of these analyses. It is not possible from the current data to state whether weight loss led to changes in weight-related affect or whether weight-related affect is causally involved in the control of subsequent eating behaviour and weight outcomes. Nonetheless, this cluster of variables has not been explored in relation to weight management before, and these relationships raise important hypotheses that should be tested prospectively. The current findings suggest that in overweight populations negative feelings about physical appearance and perceptions of social inferiority and self-criticism could potentially undermine some aspects of self-regulation of eating behaviour [22, 23, 38]. On the other hand, the ability to reassure and soothe oneself may potentially protect against weight-related negative affectivity and difficulties in regulating eating behaviour [28]. We hypothesise that if this is the case helping participants cope with the emotional stress of weight related shame and self-criticism may be a useful resource for participants engaged in weight management programmes. There is evidence that developing acceptance skills and learning to manage emotional responses has beneficial impacts in behavioural self-regulation and weight management [61, 62]. The current study suggests that addressing the role of shame and self-criticism and targeting emotion regulation through the cultivation of self-reassuring skills could enhance self-regulation of eating behaviours and improve the effectiveness of weight management programmes. However, we stress that these relationships have yet to be tested in prospective intervention studies.

### Strengths and limitations of the current study

Although these findings were supported by robust statistical analysis, namely path analyses, the cross sectional and correlational design of the study does not allow causal conclusions to be drawn. The current study tested whether a hypothesized model that links weight-focused shame, self-criticism and negative social comparisons, with difficulties with regulating eating and weight, would be consistent with the theoretically and empirically supported associations between these variables. There is growing research on the negative effect of shame and self-criticism on self-regulation of eating behaviour, but it has been mostly limited to normal weight nonclinical samples (e.g., [28, 29, 31]) or clinical samples with eating disorders (e.g., [35, 36, 39]). To our knowledge, this is the first study that tested the plausibility of this specific hypothetical model in overweight and obese participants of a community-based weight management programme. Even though cross-sectional data does not invalidate our approach (e.g., [63, 64]), future prospective studies are necessary to ascribe causality to these associations.

Another important limitation of this study was that data was collected online. Although online surveys enable the access to a large number of participants and offer a sense of privacy that facilitates the honest disclosure of sensitive data (e.g., aspects related to shame), this methodology has a number of limitations, including sampling bias (see below), self-selection issues, or under-representation of the population, and thus limits the possibility to make generalizations about the study's findings [65]. The current study used a large sample that represents individuals attending weight management programmes, but participants were predominately middle-aged, Caucasian women. Only 1.8% of the respondents were men, while about 5% of the regular membership of commercial weight management organisations comprise men and so they appear to have been under-represented in this sample. For this reason, the study was conducted only in the female participants’ sample, but future research should investigate this model considering also men. As with most surveys of this type, only a small percentage of participants in the programme who had accessed the website, actually took part in the survey. The site is accessed by >100, 000 participants per week (although the number accessing the survey description was not recorded). In a separate online study where participant access was recorded from the same population of participants of a commercial weight management programme, we have found that 10,483 participants accessed a survey, of whom 2492 completed it. There was evidence that these were relatively successful participants as on average, they had lost 10.19% of their initial weight in approximately 9 months prior to the survey. By definition of taking part in the study they were prepared to discuss their emotions in relation to their weight control. It may well be that the variables of interest present differently in those who are less successful participants in weight management programmes. This study also suggests that issues related to external shame, self-criticism, unfavourable social comparison, and low self-reassurance are important in the lives of the general public struggling with weight management and not just clinical samples with eating disorders (e.g., [35, 36, 39]). Future studies should investigate whether the pattern of associations found in the current study is stable in other samples of participants who show more difficulties in losing weight and/or maintaining weight loss.

## Supporting Information

S1 Dataset Data set used in the analysis for this study. (SAV)

S1 File Supplementary material- scales used in the survey. (DOCX)
