# Supplementary material for: The Impact of Shame, Self-Criticism and Social Rank on Eating Behaviours in Overweight and Obese Women Participating in a Weight Management Programme
Source: PLoS One. 2017 Jan 20;12(1):e0167571. doi: 10.1371/journal.pone.0167571 (PMC5249118; doi:10.1371/journal.pone.0167571)
Supplement: S1 File — (DOCX) [file pone.0167571.s002.docx]

**Weight focused Self-Criticising & Self-Reassuring Scale**

When we think about our weight, body shape and eating we can sometimes have *negative and self-critical thoughts and feelings about ourselves,* while at other times we can be *caring and supportive* of ourselves. Below are a series of thoughts and feelings that you may have experienced. Read each statement carefully and circle the number that best describes how much each statement is true for you.

*Please use the scale below.*

| Not at all  like me  0 | A little bit  like me  1 | Moderately  like me  2 | Quite a bit  like me  3 | Extremely  like me  4 |
| --- | --- | --- | --- | --- |

**When it comes to my eating and weight:**

| 1. | I am easily disappointed with myself. | 0 | 1 | 2 | 3 | 4 |
| --- | --- | --- | --- | --- | --- | --- |
| 2. | There is a part of me that puts me down. | 0 | 1 | 2 | 3 | 4 |
| 3. | I am able to remind myself of positive things about myself. | 0 | 1 | 2 | 3 | 4 |
| 4. | I find it difficult to control my anger and frustration at myself. | 0 | 1 | 2 | 3 | 4 |
| 5. | I find it easy to forgive myself. | 0 | 1 | 2 | 3 | 4 |
| 6. | There is a part of me that feels I am not good enough. | 0 | 1 | 2 | 3 | 4 |
| 7. | I feel beaten down by my own self-critical thoughts. | 0 | 1 | 2 | 3 | 4 |
| 8. | I still like being me. | 0 | 1 | 2 | 3 | 4 |
| 9. | I have become so angry with myself that I want to hurt or injure myself. | 0 | 1 | 2 | 3 | 4 |
| 10. | I have a sense of disgust with myself. | 0 | 1 | 2 | 3 | 4 |
| 11. | I can still feel lovable and acceptable. | 0 | 1 | 2 | 3 | 4 |
| 12. | I stop caring about myself. | 0 | 1 | 2 | 3 | 4 |
| 13. | I find it easy to like myself. | 0 | 1 | 2 | 3 | 4 |
| 14. | I remember and dwell on my failings. | 0 | 1 | 2 | 3 | 4 |
| 15. | I call myself names. | 0 | 1 | 2 | 3 | 4 |
| 16. | I am gentle and supportive with myself. | 0 | 1 | 2 | 3 | 4 |
| 17. | I can’t accept failures and setbacks without feeling inadequate. | 0 | 1 | 2 | 3 | 4 |
| 18. | I think I deserve my self-criticism. | 0 | 1 | 2 | 3 | 4 |
| 19. | I am able to care and look after myself. | 0 | 1 | 2 | 3 | 4 |
| 20. | There is a part of me that wants to get rid of the bits I don’t like. | 0 | 1 | 2 | 3 | 4 |
| 21. | I encourage myself for the future. | 0 | 1 | 2 | 3 | 4 |
| 22. | I do not like being me. | 0 | 1 | 2 | 3 | 4 |

**WEIGHT FOCUSED EXTERNAL SHAME (WFES)**

When we think about our weight, body shape and eating we can feel that other people might rather look down on us. With this in mind circle the number to the right of the item that indicates the frequency with which you find yourself feeling or experiencing what is described in the statement. Use the scale below.

**0 = NEVER 1 = SELDOM 2 = SOMETIMES 3 = FREQUENTLY 4 = ALMOST ALWAYS**

#

**1.** I feel other people see me as not good enough. 0 1 2 3 4

**2.** I think that other people look down on me. 0 1 2 3 4

**3.** Other people put me down a lot. 0 1 2 3 4

**4.** I feel insecure about others opinions of me. 0 1 2 3 4

**5.** Other people see me as not measuring up to them. 0 1 2 3 4

**6.** Other people see me as small and insignificant. 0 1 2 3 4

**7.** Other people see me as somehow defective as a person. 0 1 2 3 4

**8.** People see me as unimportant compared to others. 0 1 2 3 4

**9.** Other people look for my faults. 0 1 2 3 4

**10.** People see me as striving for perfection but being unable 0 1 2 3 4

to reach my own standards.

**11.** I think others are able to see my defects. 0 1 2 3 4

**12.** Others are critical or punishing when I make a mistake. 0 1 2 3 4

**13.** People distance themselves from me when I make mistakes. 0 1 2 3 4

**14.** Other people always remember my mistakes. 0 1 2 3 4

**15.** Others see me as fragile. 0 1 2 3 4

**16.** Others see me as empty and unfulfilled. 0 1 2 3 4

**17.** Others think there is something missing in me. 0 1 2 3 4

**18.** Other people think I have lost control over my body 0 1 2 3 4 and feelings.

**WEIGHT-FOCUSED SOCIAL COMPARISON SCALE**

When we think about our weight, body shape or eating we may compare ourselves with others Please circle a number at a point which best describes the way in which you see yourself in **comparison to others on the these issues**.

*For example:*

| Short |  | 1 | 2 | 3 | 4 | 5 | 6 | 7 | 8 | 9 | 10 |  | Tall |
| --- | --- | --- | --- | --- | --- | --- | --- | --- | --- | --- | --- | --- | --- |

If you put a mark at 3 this means you see yourself as shorter than others; if you put a mark at 5 (middle) about average; and a mark at 7 somewhat taller.

Circle one number on each line according to how you see yourself in relationship to others.

**Thinking about my weight and eating - in relationship to others I feel:**

| Inferior |  | 1 | 2 | 3 | 4 | 5 | 6 | 7 | 8 | 9 | 10 |  | Superior |
| --- | --- | --- | --- | --- | --- | --- | --- | --- | --- | --- | --- | --- | --- |
| Incompetent |  | 1 | 2 | 3 | 4 | 5 | 6 | 7 | 8 | 9 | 10 |  | More competent |
| Unlikeable |  | 1 | 2 | 3 | 4 | 5 | 6 | 7 | 8 | 9 | 10 |  | More likeable |
| Left out |  | 1 | 2 | 3 | 4 | 5 | 6 | 7 | 8 | 9 | 10 |  | Accepted |
| Different |  | 1 | 2 | 3 | 4 | 5 | 6 | 7 | 8 | 9 | 10 |  | Same |
| Untalented |  | 1 | 2 | 3 | 4 | 5 | 6 | 7 | 8 | 9 | 10 |  | More talented |
| Weaker |  | 1 | 2 | 3 | 4 | 5 | 6 | 7 | 8 | 9 | 10 |  | Stronger |
| Unconfident |  | 1 | 2 | 3 | 4 | 5 | 6 | 7 | 8 | 9 | 10 |  | More confident |
| Undesirable |  | 1 | 2 | 3 | 4 | 5 | 6 | 7 | 8 | 9 | 10 |  | More desirable |
| Unattractive |  | 1 | 2 | 3 | 4 | 5 | 6 | 7 | 8 | 9 | 10 |  | More attractive |
| An outsider |  | 1 | 2 | 3 | 4 | 5 | 6 | 7 | 8 | 9 | 10 |  | An insider |

**Weight focused Feeling scale**

When we think about our weight, body shape and eating we can sometimes have different feelings Below are a series feelings that you may have experienced. Read each statement carefully and circle the number that best describes how much each statement is true for you.

*Please use the scale below.*

| Not at all  like me  0 | A little bit  like me  1 | Moderately  like me  2 | Quite a bit  like me  3 | Extremely  like me  4 |
| --- | --- | --- | --- | --- |

**When I think about my eating and weight:**

| 1. | I am angry that I am like this | 0 | 1 | 2 | 3 | 4 |
| --- | --- | --- | --- | --- | --- | --- |
| 2. | I feel depressed and down | 0 | 1 | 2 | 3 | 4 |
| 3. | I am quite happy in myself. | 0 | 1 | 2 | 3 | 4 |
| 4. | I am anxious about myself. | 0 | 1 | 2 | 3 | 4 |
| 5. | I am irritable. | 0 | 1 | 2 | 3 | 4 |
| 6. | I am pessimistic I can change. | 0 | 1 | 2 | 3 | 4 |
| 7. | I feel frustrated | 0 | 1 | 2 | 3 | 4 |
| 8. | I feel pleased with my efforts | 0 | 1 | 2 | 3 | 4 |
| 9. | I feel weak. | 0 | 1 | 2 | 3 | 4 |
| 10. | I feel it is not that important | 0 | 1 | 2 | 3 | 4 |
| 11. | I feel disgusted with myself | 0 | 1 | 2 | 3 | 4 |
| 12. | I feel good with myself. | 0 | 1 | 2 | 3 | 4 |

| **DASS21**  Please read each statement and circle a number 0, 1, 2 or 3 which indicates how much the statement applied to you *over the past week*. There are no right or wrong answers. Do not spend too much time on any statement.  *The rating scale is as follows:*   1. Did not apply to me at all 2. Applied to me to some degree, or some of the time 3. Applied to me to a considerable degree, or a good part of time 4. Applied to me very much, or most of the time | | | | | |
| --- | --- | --- | --- | --- | --- |
| 1 | I found it hard to wind down | 0 | 1 | 2 | 3 |
| 2 | I was aware of dryness of my mouth | 0 | 1 | 2 | 3 |
| 3 | I couldn’t seem to experience any positive feeling at all | 0 | 1 | 2 | 3 |
| 4 | I experienced breathing difficulty (eg, excessively rapid breathing, breathlessness in the absence of physical exertion) | 0 | 1 | 2 | 3 |
| 5 | I found it difficult to work up the initiative to do things | 0 | 1 | 2 | 3 |
| 6 | I tended to over-react to situations | 0 | 1 | 2 | 3 |
| 7 | I experienced trembling (eg, in the hands) | 0 | 1 | 2 | 3 |
| 8 | I felt that I was using a lot of nervous energy | 0 | 1 | 2 | 3 |
| 9 | I was worried about situations in which I might panic and make a fool of myself | 0 | 1 | 2 | 3 |
| 10 | I felt that I had nothing to look forward to | 0 | 1 | 2 | 3 |
| 11 | I found myself getting agitated | 0 | 1 | 2 | 3 |
| 12 | I found it difficult to relax | 0 | 1 | 2 | 3 |
| 13 | I felt down-hearted and blue | 0 | 1 | 2 | 3 |
| 14 | I was intolerant of anything that kept me from getting on with what I was doing | 0 | 1 | 2 | 3 |
| 15 | I felt I was close to panic | 0 | 1 | 2 | 3 |
| 16 | I was unable to become enthusiastic about anything | 0 | 1 | 2 | 3 |
| 17 | I felt I wasn’t worth much as a person | 0 | 1 | 2 | 3 |
| 18 | I felt that I was rather touchy | 0 | 1 | 2 | 3 |
| 19 | I was aware of the action of my heart in the absence of physical exertion (eg, sense of heart rate increase, heart missing a beat) | 0 | 1 | 2 | 3 |
| 20 | I felt scared without good reason | 0 | 1 | 2 | 3 |
| 21 | I felt that life was meaningless | 0 | 1 | 2 | 3 |

## Three factor eating questionnaire

The following statements describe certain behaviours or feelings. Please mark whether these statements apply to yourself or not. If you have the feeling that a statement is neither completely true nor false for yourself, please mark the answer that describes your personal situation as best as possible.

1. When I smell a sizzling steak or see a juicy peace of meat, I find true ⭘ false ⭘

it very difficult to keep from eating, even if I have just finished a meal

1. I usually eat too much at social occasions, like parties and picnics true ⭘ false ⭘
2. I am usually so hungry that I eat more than three times a day true ⭘ false ⭘
3. When I have eaten my quota of calories, I am usually good about not true ⭘ false ⭘

eating any more

1. Dieting is so hard for me because I just get too hungry true ⭘ false ⭘
2. I deliberately take small helpings as a means of controlling my weight true ⭘ false ⭘
3. Sometimes things just taste so good that I keep on eating even when I true ⭘ false ⭘

am no longer hungry

1. Since I am often hungry, I sometimes wish that while I am eating, an true ⭘ false ⭘

expert would tell me that I have had enough or that I can have

something more to eat.

1. When I feel anxious, I find myself eating. true ⭘ false ⭘
2. Life is too short to worry about dieting true ⭘ false ⭘
3. Since my weight goes up and down, I have gone on reducing diets true ⭘ false ⭘

more than once

1. I often feel so hungry that I just have to eat something true ⭘ false ⭘
2. When I am with someone who is overeating, I usually overeat too. true ⭘ false ⭘
3. I have a pretty good idea of the number of calories in common food. true ⭘ false ⭘
4. Sometimes when I start eating, I just can’t seem to stop true ⭘ false ⭘
5. It is not difficult for me to leave something on my plate true ⭘ false ⭘
6. At certain times of the day, I get hungry because I have gotten used to true ⭘ false ⭘

eating then.

1. While on a diet, if I eat food that is not allowed, I consciously eat less true ⭘ false ⭘

for a period of time to make up for it.

1. Being with someone who is eating often makes me hungry enough to true ⭘ false ⭘

eat also

1. When I feel blue, I often overeat. true ⭘ false ⭘
2. I enjoy eating too much to spoil it by counting calories or watching my true ⭘ false ⭘

weight

1. When I see a real delicacy, I often get so hungry that I have to eat it true ⭘ false ⭘

right away

1. I often stop eating when I am not really full as a conscious means of true ⭘ false ⭘

limiting the amount that I eat.

1. I get so hungry that my stomach often seems like a bottomless pit true ⭘ false ⭘
2. My weight has hardly changed at all in the last ten years true ⭘ false ⭘
3. I am always hungry so it is hard for me to stop eating before I finish true ⭘ false ⭘

the food on my plate

1. When I feel lonely, I console myself by eating true ⭘ false ⭘
2. I consciously hold back at meals in order not to gain weight true ⭘ false ⭘
3. I sometimes get very hungry late in the evening or at night. true ⭘ false ⭘
4. I eat anything I want, any time I want. true ⭘ false ⭘
5. Without even thinking about it, I take a long time to eat. true ⭘ false ⭘
6. I watch portion sizes as a conscious means of controlling my weight. true ⭘ false ⭘
7. I do not eat some foods because they make me fat. true ⭘ false ⭘
8. I am always hungry enough to eat at any time. true ⭘ false ⭘
9. I pay a great deal of attention to changes in my figure. true ⭘ false ⭘
10. While on a diet, if I eat a food that is not allowed, I often then splurge true ⭘ false ⭘

and eat other high calorie foods.

*Please answer the following questions by marking the response that is appropriate to you.*

1. How often are you dieting in a conscious effort to control your weight?

| ⭘ | ⭘ | ⭘ | ⭘ |
| --- | --- | --- | --- |
| rarely | sometimes | usually | always |

1. Would a weight fluctuation of 5 lbs / 2.5kg affect the way you live your life?

| ⭘ | ⭘ | ⭘ | ⭘ |
| --- | --- | --- | --- |
| not at all | slightly | moderately | very much |

1. How often do you feel hungry?

| ⭘ | ⭘ | ⭘ | ⭘ |
| --- | --- | --- | --- |
| only at mealtimes | sometimes between meals | often between meals | almost always |

1. Do your feelings of guilt about overeating help you to control you food intake?

| ⭘ | ⭘ | ⭘ | ⭘ |
| --- | --- | --- | --- |
| never | rarely | often | always |

1. How difficult would it be for you to stop eating halfway through dinner and not eat for the next four hours?

| ⭘ | ⭘ | ⭘ | ⭘ |
| --- | --- | --- | --- |
| easy | slightly difficult | moderately difficult | very difficult |

1. How conscious are you of what you are eating?

| ⭘ | ⭘ | ⭘ | ⭘ |
| --- | --- | --- | --- |
| not at all | slightly | moderately | extremely |

1. How frequently do you avoid ‘stocking up’ on tempting foods?

| ⭘ | ⭘ | ⭘ | ⭘ |
| --- | --- | --- | --- |
| almost never | seldom | usually | almost always |

1. How likely are you to shop for low calorie foods?

| ⭘ | ⭘ | ⭘ | ⭘ |
| --- | --- | --- | --- |
| unlikely | slightly likely | moderately likely | very likely |

1. Do you eat sensibly in front of others and splurge alone?

| ⭘ | ⭘ | ⭘ | ⭘ |
| --- | --- | --- | --- |
| never | rarely | often | always |

1. How likely are you to consciously eat slowly in order to cut down on how much you eat?

| ⭘ | ⭘ | ⭘ | ⭘ |
| --- | --- | --- | --- |
| unlikely | slightly likely | moderately likely | very likely |

1. How frequently do you skip dessert because you are no longer hungry?

| ⭘ | ⭘ | ⭘ | ⭘ |
| --- | --- | --- | --- |
| never | seldom | at least once a week | almost every day |

1. How likely are you to consciously eat less than you want?

| ⭘ | ⭘ | ⭘ | ⭘ |
| --- | --- | --- | --- |
| unlikely | slightly likely | moderately likely | very likely |

1. Do you go on eating binges though you are not hungry?

| ⭘ | ⭘ | ⭘ | ⭘ |
| --- | --- | --- | --- |
| never | rarely | sometimes | at least once a week |

1. On a scale of 0 to 5, where 0 means no restraint in eating (eating whatever your want, whenever you want it and 5 means total restraint (constantly limiting food intake and never ‘giving in’), what number would you give yourself?

0 = eat whatever you want, whenever you want it ⭘

1 = usually eat whatever you want, whenever you want it ⭘

2 = often eat whatever you want, whenever you want it ⭘

3 = often limit food intake, but often ‘give in’ ⭘

4 = usually limit food intake, rarely ‘give in’ ⭘

5 = constantly limiting food intake, never ‘giving in’ ⭘

1. To what extend does this statement describe your eating behaviour? ‘I start dieting in the morning, but because of any number of things that happen during the day, by the evening I have given up and eat, what I want, promising myself to start dieting again tomorrow’

| ⭘ | ⭘ | ⭘ | ⭘ |
| --- | --- | --- | --- |
| not like me | little like me | pretty good  description of me | describes me  perfectly |

**The Warwick-Edinburgh Mental Well-being Scale**

**(WEMWBS)**

**Below are some statements about feelings and thoughts.**

**Please tick the box that best describes your experience of each over the last 2 weeks**

| **STATEMENTS** | **None of the time** | **Rarely** | **Some of the time** | **Often** | **All of the time** |
| --- | --- | --- | --- | --- | --- |
| I’ve been feeling optimistic about the future | **1** | **2** | **3** | **4** | **5** |
| I’ve been feeling useful | **1** | **2** | **3** | **4** | **5** |
| I’ve been feeling relaxed | **1** | **2** | **3** | **4** | **5** |
| I’ve been feeling interested in other people | **1** | **2** | **3** | **4** | **5** |
| I’ve had energy to spare | **1** | **2** | **3** | **4** | **5** |
| I’ve been dealing with problems well | **1** | **2** | **3** | **4** | **5** |
| I’ve been thinking clearly | **1** | **2** | **3** | **4** | **5** |
| I’ve been feeling good about myself | **1** | **2** | **3** | **4** | **5** |
| I’ve been feeling close to other people | **1** | **2** | **3** | **4** | **5** |
| I’ve been feeling confident | **1** | **2** | **3** | **4** | **5** |
| I’ve been able to make up my own mind about things | **1** | **2** | **3** | **4** | **5** |
| I’ve been feeling loved | **1** | **2** | **3** | **4** | **5** |
| I’ve been interested in new things | **1** | **2** | **3** | **4** | **5** |
| I’ve been feeling cheerful | **1** | **2** | **3** | **4** | **5** |
